# Women's Health Decision-Making Autonomy and Skilled Birth Attendance in Ghana

**DOI:** 10.1155/2016/6569514

**Published:** 2016-12-26

**Authors:** Edward Kwabena Ameyaw, Augustine Tanle, Kwaku Kissah-Korsah, Joshua Amo-Adjei

**Affiliations:** ^1^Department of Population and Health, Faculty of Social Sciences, University of Cape Coast, Cape Coast, Ghana; ^2^African Population and Health Research Centre, Nairobi, Kenya

## Abstract

Delivering in health facility under the supervision of skilled birth attendant is an important way of mitigating impacts of delivery complications. Empirical evidence suggests that decision-making autonomy is aligned with holistic wellbeing especially in the aspect of maternal and child health. The objective of this paper was to examine the relationship between women's health decision-making autonomy and place of delivery in Ghana. We extracted data from the 2014 Ghana Demographic and Health Survey. Descriptive and logistic regression techniques were applied. The results indicated that women with health decision-making autonomy have higher tendency of health facility delivery as compared to those who are not autonomous [OR = 1.27, CI = 1.09–1.48]. However, those who have final say on household large purchases [OR = 0.71, CI = 0.59–0.84] and those having final say on visits [OR = 0.86, CI = 0.73–1.01] were less probable to deliver in health facility than those without such decision-making autonomy. Consistent with existing evidence, wealthier, urban, and highly educated women had higher inclination of health facility delivery. This study has stressed the need for interventions aimed at enhancing health facility delivery to target women without health decision-making autonomy and women with low education and wealth status, as this can play essential role in enhancing health facility delivery.

## 1. Introduction

The World Health Organisation (WHO) estimates that 536,000 women die yearly owing to pregnancy and delivery related complications. At the same time, over the past two decades, maternal mortality ratio has declined by 45 percent whilst prevalence of maternal death declined from 532,000 to 289,000 as of 2015 [1]. Despite the general reduction, disparities persist between developed and developing countries. Whereas developed countries have virtually overcome this problem; low and middle income countries account for about 99.0 percent of all maternal and child deaths [2, 3]. Ghana as a developing country is similarly faced with high maternal mortality rate although some progress has been chalked. Between 1990 and 2013, maternal mortality rate dropped from 760 to 380 per 100,000 live births whilst it stood at 319 by 2015 which was far above the MDG 5 target of 190 [4]. Considering the current trend, Ghana is projected to achieve the 2015 MDG 5 target in 2037. Skilled birth attendance (SBA) has been identified as an effective mechanism for mitigating the risks of maternal deaths during delivery [5]. It encompasses care offered by accredited health professionals during labour, birth, and postnatal period. SBA offers safety and life sustaining environment for both mothers and newborns thereby reducing chances of complications [5].

Despite the substantial benefits of SBA to maternal health, 40 million women of 115 million who became pregnant in 2013 delivered at home without skilled birth attendants. These deliveries have high risks of complications that can lead to avoidable maternal morbidity and mortality [6]. Direct causes of maternal death such as haemorrhage and sepsis can be averted or significantly reduced by adequate SBA [7]. For instance, in 2015, developing countries accounted for nearly 99% (302,000) of global maternal deaths with 66% (201,000) occurring in sub-Saharan Africa [6].

Decision-making on place of delivery particularly in the developing world is not only the prerogative of women but shaped by several actors and factors, some of which are beyond the remit of women. Enhancing empowerment status of women in developing countries constitutes one of the recognised approaches to improving maternal healthcare service utilisation [8]. Studies have demonstrated that decision-making capability of women affects healthcare utilisation through various pathways. Analysis of gender inequities in women's access to reproductive health services between sub-Saharan African and South Asia found that women's abilities to control earnings and influence household decision-making particularly about healthcare are positive predictors for maternal healthcare utilisation [9–11]. Similar observations have also been made in Ethiopia and Tajikistan on the positive effects of women's decision-making autonomy on their physical mobility [11, 12].

Some evidence from northern Ghana indicates that pathways through which empowerment enhances healthcare utilisation include but are not limited to income, education, possession of assets, liberation from domestic violence, and liberty to move without restrictions. However, a combination of these factors is required for women to have a final say about issues pertaining to their healthcare utilisation [13, 14]. At the same time, it has been noted that low empowerment status curtails women's ability to access healthcare; for instance, an assessment of participation in Community-based Health Planning and Services (CHPS) in Ghana indicated that male dominance and limited women's access to services are rendered by the CHPS compounds [15].

Also, studies have revealed that the suppression of women manifests in diverse ways including marital rape, disparity in employment, and low participation in decision-making [16–20]. The Millennium Development Goals report of the country indicated that the quest to promote gender equality and empower women (MDG 3) and improve maternal health (MDG 5) could not be achieved [21] whilst maternal mortality rate stood at 144 per 100,000 live births as of 2015 which far exceeds the global target of 54 per 100,000 live births [21]. At the same time, health facility-based delivery stands at 69% [22].

Previous studies have reported inconsistent effects of women's decision-making autonomy on the use of health services [11, 13–15]. This may be due to the fact that virtually all the existing studies aggregate three autonomy variables—control over finance, decision-making power, and extent of freedom of movement. We argue in this paper that aggregating all these decision variables can deflate/conflate the net effects of each of the variables popularly used to examine women's empowerment and their health behaviours and practices. This study extends the evidence by examining effects of women's health decision-making power net of other socioeconomic determinants and how it shapes their utilisation of health facilities for delivery. As a result, this study seeks to explore how women's healthcare decision-making autonomy affects place of delivery in Ghana.

## 2. Methods

### 2.1. Study Setting

The study setting is Ghana which is centrally located on the West African coast and has a total land area of 238,537 km^2^. It lies about 750 km north of the equator between latitudes 4° North and 12° North as well as longitudes 4° West and 2° East [23]. According to the 2010 Population and Housing Census (PHC), the country's population was 24,658,823 [23].

Additionally, diverse initiatives have been initiated to further improve universal healthcare and maternal health in the country such as the implementation of free delivery policy as well as development of the National Newborn Health Strategy and Action Plan [24]. Prior to these, Community Health Planning and Services (CHPS) was instituted in 2000 as a means of rendering a close-to-client service where community health nurses are posted to communities to render public health and limited clinical services and serve as first point of call and referrals.

Health service provision in Ghana is ordered at three levels, namely, primary, secondary, and tertiary, with their corresponding levels of management as national/central headquarters, regional, and district. Private health institutions offer about 40% of health services in the country and they include not-for-profit organisations such as the Christian Health Association of Ghana [24]. To minimise the cost of health services, the government in 2004 introduced a National Health Insurance Scheme under which maternal health and child health were also prioritised [25, 26]. The 2014 Ghana Demographic and Health Survey (GDHS) indicated that 51 percent and 38 percent men and women, respectively, had subscribed to the National Health Insurance Scheme (NHIS) signifying a sharp decline from what was recorded in 2008 GHDS (60% and 70.0% women and men, resp.) [26, 27].

### 2.2. Data Source

The women file from the GDHS sixth round (2014 GDHS) was used for this study. The Demographic and Health Survey (DHS) captures data on various aspects of women empowerment and maternal health conditions within the country and therefore was considered suitable for this study. Although the sample size for the study constituted 9,396 women (aged 15–49) from 11,835 households nationwide, 5,884 had delivered within the five-year period preceding the survey [27]. The 2014 GDHS was conducted with an updated frame from the 2010 Population and Housing Census prepared by the Ghana Statistical Service (GSS). The survey constituted two-stage sample design for the purpose of allowing estimates of core indicators at the national level. The initial phase constituted selection of sample points (clusters) involving enumeration areas (EAs) outlined for the 2010 PHC in which 427 clusters were designated constituting 216 from urban and 211 from rural areas. The next stage utilised systematic sampling of households in which household inventory operation was conducted in all the identified EAs. Afterwards, the households to be considered for the survey were selected from the list randomly [27].

### 2.3. Description of Variables

The outcome variable was place of delivery whilst the independent variable was healthcare decision-making autonomy of women. The standard DHS indicator for measuring women's health decision-making autonomy is a woman's ability to decide on her own healthcare [27]. With this, autonomous woman was considered as someone who decides on healthcare alone coded as “0” whilst anyone taking health decision with others was considered as not autonomous coded as “1.” This is because a couple of studies have revealed low decision-making participation among Ghanaian women [16, 18] and therefore it is suitable for someone to be considered to have healthcare decision-making autonomy if she can decide alone on her own healthcare. Some variables were recoded for simplicity of analysis and presentation. Specifically, marital status was recoded into not married = 0 and married = 1; occupation was recoded as not working = 0, primary = 1, secondary = 2, and tertiary = 3, and religious affiliation was recoded as Christianity = 1, Islam = 2, Traditionalist = 3, and no religion = 4. Partner's occupation was recoded as primary = 0, secondary = 1, and tertiary = 2.

### 2.4. Analytical Approach

The study employed STATA (version 13.1) to analyse the data. Descriptive and logistic regression techniques were applied since the outcome variable was dichotomous. Three models were developed in all. The first model presents analysis of the explanatory variable and outcome variable alone in order to determine the independent effect of the explanatory variable on the outcome variable. The second model factored other two variables for determining women's autonomy; these were having a final say on household large purchases and visits to relatives for the purpose of exploring how they induce delivery in health facility. Finally, the third model controlled for other sociodemographic variables that have the tendency to influence utilisation of SBA. The results were weighted with the available sample weight factor within the GHDS data in order to reduce sampling bias. The complex design of the survey was also factored into the data analysis.

## 3. Results

### 3.1. Descriptive Results

The ages of the respondents ranged between 15 and 49 (mean = 29.7; SD = 9.713). About half of the respondents had attained secondary education (48%) whilst a greater proportion of them were engaged in tertiary occupation (43%) as indicated in Table 1. The majority (76%) of women were affiliated to Christianity; most of the respondents reported being married (63%) with more than one-third (43%) in poor households (Table 1).

The study revealed that a greater share of Ghanaian women in the reproductive age are not autonomous when it comes to decision-making on their healthcare (78.1%). The same observation was made with regard to large household purchases as 81.4 percent were not autonomous whilst at the same time a greater proportion did not have autonomy in deciding alone on visiting family members (78.5%) as indicated in Table 2.

As indicated in Table 3, 7 out of 10 with healthcare decision-making autonomy delivered in health facilities (71.9%) whilst 67.3 percent of women without healthcare decision-making autonomy delivered in health facilities. Women aged 45–49 recorded the highest share of home deliveries (43%) whilst those aged 15–19 recorded the highest health facility delivery (77.4%). Analysed by education, a significantly higher proportion of health facility delivery occurred among women with higher/tertiary educational attainment (98%) (Table 3).

On the basis of residential status, health facility delivery was highest among urban women (91%) compared to rural women (58%) (Table 3). Health facility delivery among NHIS subscribers was higher (77%) than for nonsubscribers (66.0%). As mediums of mass media, television, radio, and newspapers media might be prominent sources of education to women by exposing them to the merits and demerits associated with home and health facility deliveries (Table 3).

### 3.2. Logistic Regression Results

In the logistic regression model, we found that women having a final say on their own healthcare (health decision-making autonomy) were more likely to deliver in health facilities as compared to those who take such decision with others [OR = 1.27, CI = 1.09–1.48] and this persisted even after controlling for the sociodemographic variables [OR = 1.20, CI = 0.98–1.48] as displayed in Table 4. However, women with final say on household large purchases and visits were less likely to utilise health facility for delivery [OR = 0.71, CI = 0.59–0.84 and OR = 0.86, OR = 0.73–1.01 resp.] (Table 4). Higher odds of health facility delivery were associated with each higher category of wealth. Specifically, women in the richest quintile [OR = 5.95, CI = 3.19–11.10] were more inclined to deliver in a health facility than poorest women (Table 4).

Women in all age categories were less probable to deliver in health facilities as compared to those aged 15–19 years (Table 4). The model revealed that women with higher educational attainment [OR = 19.39, CI = 2.67–140.94] were far more probable to deliver in health facilities as compared to those without education. On religious affiliation, it was noted that Christians and Moslems had higher odds of delivering in health facilities [OR = 1.99, CI = 1.43–2.78 and OR = 2.15, CI = 1.49–3.09 resp.] as compared to women without any religious affiliation as indicated in Table 4. The model further revealed that married women were more likely to deliver in health facilities as compared to unmarried women [OR = 1.11, CI = 0.93–1.34].

Again urban residents were noted to have higher odds of health facility delivery as compared to their rural counterparts [OR = 2.23, CI = 1.78–2.78]. Women who had subscribed to the NHIS were more likely to deliver in health facilities compared with unsubscribed counterparts [OR = 1.64, CI = 1.39–1.92]. With the Western region as a reference, women from Upper East stood out as having five times likelihood of health facility delivery [OR = 5.01, CI = 3.34–7.51] as displayed in Table 4.

## 4. Discussion

Previous studies have demonstrated that some women in Ghana are less empowered and have limited decision-making rights. This study was carried out to examine the relationship between women's health decision-making power on their place of delivery.

It was revealed that women with autonomous health decision-making power were more likely to utilise health facility for delivery as compared to those without. This presupposes that autonomy of women in relation to health is important to enhancing health facility delivery which guarantees skilled birth attendance. To a greater extent maternal health and child health are inherent in the level of empowerment and autonomy exercised by women because autonomous women would make right choices and access quality healthcare at all times particularly during delivery.

This observation is consistent with some prior studies which also noted that when women are autonomous and have much influence on decisions that affect their wellbeing, their access to and utilisation of healthcare improves [7–9]. As women usually serve as primary caregivers, when they are able to make critical decisions such as health, better outcomes will be realised as indicated in this study. As a result, enhancing empowerment status of women in developing countries constitutes one of the recognised approaches to improving maternal healthcare utilisation [6].

Delivery in health facilities increased with higher wealth quintile whereas home deliveries characterised poor women. This finding highlights the influence of cost on utilisation of healthcare in the country and this might pose threat to the health status and maternal wellbeing of poor women. This is particularly critical because cost can also deter them from accessing quality healthcare for themselves and their children even after delivery and this might constitute severe threat to their health status and their children. Similar findings were noted in a study conducted in Nepal where it was realised that home delivery was dominant among poorer than wealthier women [26] whilst another study in Ghana also noted that wealth induces maternal healthcare utilisation [27].

In line with wealth status and utilisation of health facilities, health insurance is a cost cutting intervention that enables people to have easy access to healthcare. The finding that insured women had high propensity of delivering in health facilities indicates that indeed subscription to the insurance scheme immensely influences decision on place of delivery. This observation conforms to a study conducted in the Ga-East Municipality of Ghana which noted higher rate of health facility delivery among NHIS subscribers [28].

It was observed that respondents aged 15–19 years were more probable to deliver in health facilities as compared to those aged 45–49 in Ghana. Maternal age has been linked to parity and various aspects of life [29], realising that older women have higher propensity of home delivery may be due to negative experiences during previous deliveries in health facilities such as attitude of care providers [30]. This observation might also be due to the thought that they have much experience as far as delivery is concerned and therefore there is no need to seek SBA or health facility. Ironically, maternal mortality is higher among higher parity women [5] and this necessitates the need to give important consideration and reach out to higher parity women to increased use of SBA.

It was noted that higher educational attainment was highly related to health facility delivery which affirms the plethora of studies [6–8] that have made the same findings. This presupposes that as women ascend the education ladder, they gain much insight into the need to deliver in health facilities which their uneducated counterparts may be oblivious of as noted by some prior studies [31, 32].

The study has indicated that health facility delivery is more probable among urban residents as compared to those in rural settings. This might be owing to the relatively less availability of health facilities in rural settings as compared to urban areas in the country. If a woman in rural area has to ply several miles before accessing the nearest health facility, such a person might not have the urge to consistently access health facility as compared to those in urban areas where health facilities relatively abound. At the same time, urban areas have relatively better road networks, which enhances their easy to access health facilities for deliveries. Analysis of trend and pattern of institutional delivery in Bangladesh from 1993 to 2007 in relation to residency and education also revealed low utilisation of health facility among women in rural settings [33]. On the contrary, a Nigerian study [34] noted that most urban dwellers deliver at home. This disparity might be attributable to variation in other factors prevailing in these two different study areas such as attitude of healthcare providers.

Women in the Upper East region were noted to have the highest likelihood of health facility delivery in the country. The high presence of CHPS in the region may be contributing substantially to the observed pattern. The relevance of CHPS in improving maternal and child health in Ghana has been highlighted previously [35, 36], suggesting that physical access is equally an important drive towards facility-based deliveries.

Since the study dwelt on preexisting data, it could not explore the motivation for the use of place of delivery among women. Again, the cross-sectional nature of the survey does not permit identifying cause and effect. Amidst all these, the results present a true reflection of the relationship between health empowerment and place of delivery and thereby provide insights into public health interventions worth instituting to promote health facility delivery in Ghana.

## 5. Conclusions

The study has indicated that Ghanaian women with healthcare decision-making autonomy are more likely to deliver in health facilities and this persisted even after controlling for sociodemographic factors. It is also evident that higher wealth status is aligned with health facility delivery. The study therefore recommends that government and nongovernmental organisations concerned with maternal and reproductive health should target women who do not independently decide on their own healthcare with mass media campaigns advocating health facility delivery. Additionally, incentive packages required to inspire health facility delivery must be geared towards women with low education and wealth status, as this can play essential role in enhancing health facility delivery.

## Figures and Tables

**Table 1:** Background characteristics, Ghana DHS 2014 (*N* = 5,884).

Background characteristics	Frequency (%)
*Age*	
15–19	211 (3.6)
20–24	1,003 (17.1)
25–29	1,468 (25.0)
30–34	1,465 (25.0)
35–39	1,087 (18.5)
40–44	504 (8.6)
45–49	146 (2.5)
*Level of education*	
No Education	1,613 (27.4)
Primary	1,179 (20.0)
Secondary	2,830 (48.1)
Higher/tertiary	262 (4.5)
*Religious affiliation*	
Christianity	4,446 (75.6)
Islam	1,002 (17.0)
Traditionalist	189 (3.2)
No religion	246 (4.2)
*Marital status*	
Married	3,722 (63.3)
Not married	2,162 (36.8)
*Occupation*	
Not working	1,035 (17.6)
Primary	1,554 (26.5)
Secondary	745 (12.7)
Tertiary	2,536 (43.2)
*Wealth status*	
Poor	2,541 (43.2)
Middle	1,151 (19.6)
Rich	2,192 (37.3)
*Residential status*	
Rural	3,236 (55.0)
Urban	2,648 (45.0)
*Region of residence*	
Western	443 (10.3)
Central	469 (11.0)
Greater Accra	698 (16.3)
Volta	325 (7.6)
Eastern	402 (9.4)
Ashanti	765 (17.9)
Brong Ahafo	387 (9.0)
Northern	497 (11.6)
Upper east	182 (4.3)
Upper west	115 (2.7)

Source: computed from 2014 GDHS data set.

**Table 2:** Empowerment status by category, Ghana DHS 2014.

Variable	Frequency	Percentage (%)
*Healthcare decision-making autonomy*		
Autonomous	1,117	21.9
Not autonomous	3,990	78.1
*Large household purchases decision-making*		
Autonomous	948	18.6
Not autonomous	4,159	81.4
*Visit to family members decision-making*		
Autonomous	1,097	21.5
Not autonomous	4,008	78.5

Source: computed from 2014 GDHS dataset.

**Table 3:** Distribution of place of delivery by healthcare decision-making autonomy.

Variable	Home Delivery (%)	Health Facility (%)
*Decision making on healthcare*		
Respondent alone	28.1	71.9
Respondent and others	32.7	67.3
*Age*		
15–19	22.6	77.4
20–24	31.0	69.0
25–29	25.2	74.9
30–34	26.6	73.4
35–39	23.5	76.6
40–44	31.0	69.0
45–49	42.9	57.2
*Wealth status*		
Poorest	54.2	45.8
Poorer	40.5	59.5
Middle	24.2	75.8
Richer	6.6	93.4
Richest	3.0	97.0
*Education*		
No education	48.7	51.3
Primary	32.1	67.9
Secondly	14.9	85.2
Higher/tertiary	2.0	98.0
*Religious affiliation*		
Christianity	22.9	77.1
Islam	28.8	71.2
Traditionalist	79.3	20.8
No religion	53.9	46.2
*Marital status*		
Not married	27.6	72.4
Married	26.8	73.2
*Residential status*		
Rural	41.6	58.4
Urban	9.4	90.6
*Region of residence*		
Western	35.3	64.7
Central	28.8	71.2
Greater Accra	32.9	67.1
Volta	38.4	61.6
Eastern	30.0	70.4
Ashanti	27.6	72.4
Brong Ahafo	31.6	68.4
Northern	32.0	68.1
Upper east	37.2	63.0
Upper west	26.3	73.7
*NHIS Subscription*		
No	34.5	65.6
Yes	23.5	76.5
*Partner's occupation*		
Primary	48.0	52.0
Secondary	16.8	83.2
Tertiary	10.8	89.2
*Frequency of watching TV*		
Not at all	45.6	54.4
Less than once a week	24.2	75.9
At least once a week	16.0	84.0
*Frequency of listening to radio*		
Not at all	47.7	52.2
Less than a week	32.1	67.9
At least once a week	23.5	76.5
*Frequency of reading newspaper*		
Not at all	33.4	66.6
Less than once a week	8.1	91.9
At least once a week	6.3	93.7

Source: computed from 2014 GDHS.

**Table 4:** Binary logistic results on relationship between decision-making autonomy and place of delivery in Ghana.

Variable	Model I	95% CI	Model II	95% CI	Model III	95% CI
*Final say on own healthcare*						
Respondent and others	1^*∗∗*^	(1,1)	1^*∗∗∗*^	(1,1)	1	(1,1)
Respondent Alone	1.27	(1.09–1.48)	1.49	(1.26–1.77)	1.20	(0.98–1.48)
*Final say on household large purchases*						
Respondent and others			1^*∗∗∗*^	(1,1)	1	(1,1)
Respondent Alone			0.71	(0.59–0.84)	0.88	(0.71–1.09)
*Final say on visits*						
Respondent and others			1	(1,1)	1	(1,1)
Respondent Alone			0.86	(0.73–1.01)	0.92	(0.74–1.13)
*Age*						
15–19					1	(1,1)
20–24					0.77	(0.45–1.31)
25–29					0.71	(0.42–1.29)
30–34					0.69	(0.41–1.18)
35–39					0.83	(0.49–1.41)
40–44					0.83	(0.47–1.44)
45–49					0.87	(0.46–1.62)
*Education*						
No education					1	(1,1)
Primary					1.33^*∗∗*^	(1.08–1.64)
Secondary					2.27^*∗∗∗*^	(1.84–2.80)
Higher/Tertiary					19.39^*∗∗*^	(2.67–140.94)
*Religious affiliation*						
No religion					1	(1,1)
Christianity					1.99^*∗∗∗*^	(1.43–2.78)
Islam					2.15^*∗∗∗*^	(1.49–3.09)
Traditionalist					0.57^*∗*^	(0.35–0.91)
*Wealth Status*						
Poorest					1	(1,1)
Poorer					1.25^*∗*^	(1.01–1.55)
Middle					1.68^*∗∗∗*^	(1.26–2.23)
Richer					4.08^*∗∗∗*^	(2.69–6.18)
Richest					5.95^*∗∗∗*^	(3.19–11.10)
*Occupation*						
Not working					1	(1,1)
Primary					1.09	(0.87–1.38)
Secondary					1.16	(0.88–1.54)
Tertiary					1.37	(1.08–1.75)
*Marital status*						
Not married					1	(1,1)
Married					1.11	(0.93–1.34)
*Residential status*						
Rural					1	(1,1)
Urban					2.23^*∗∗∗*^	(1.78–2.78)
*NHIS subscription*						
Not subscribed					1	(1,1)
Subscribed					1.64^*∗∗∗*^	(1.39–1.92)
*Region of residence*						
Western					1	(1,1)
Central					0.91	(0.65–1.27)
Greater Accra					1.37	(0.83–2.27)
Volta					0.85	(0.61–1.19)
Eastern					0.83	(0.59–1.15)
Ashanti					1.51	(1.03–2.20)
Brong Ahafo					1.72	(1.24–2.40)
Northern					0.57	(0.41–0.80)
Upper east					5.01	(3.34–7.51)
Upper west					1.61	(1.13–2.30)
*Partner's occupation*						
Primary					1	(1,1)
Secondary					1.15^*∗∗*^	(0.93–1.44)
Tertiary					1.45	(1.09–1.92)
*Frequency of watching TV*						
Not at all					1	(1,1)
Less than once a week					0.86	(0.70–1.07)
At least once a week					1.07	(0.87–1.32)
*Frequency of listening to radio*						
Not at all					1	(1,1)
Less than once a week					1.12	(0.91–1.37)
At least once a week					1.52	(1.24–1.88)
*Frequency of listening reading newspaper*						
Not at all					1	(1,1)
Less than once a week					1.37	(0.81–2.31)
At least once a week					1.02	(0.56–1.88)

Source: computed from 2014 GDHS dataset. M = Model; OR = odds ratio; *p* value in parenthesis; confidence interval in square brackets; ref = reference; ^*∗*^*p* < 0.05, ^*∗∗*^*p* < 0.01, and ^*∗∗∗*^*p* < 0.001.
